# CT imaging of third inflow cases: a hidden cause of surgical complications and liver pseudolesions

**DOI:** 10.1093/bjrcr/uaaf042

**Published:** 2025-07-26

**Authors:** Gizem Cural Kula, Afak Durur Karakaya

**Affiliations:** Faculty of Medicine, Department of Radiology, Koc University, Istanbul, 34010, Turkey; Faculty of Medicine, Department of Radiology, Koc University, Istanbul, 34010, Turkey

**Keywords:** aberrant left gastric vein, hepatic surgery, gastric surgery, third inflow, portal vein tributaries, pseudolesion

## Abstract

Third inflow refers to the additional blood supply to the liver from a third source, apart from its dual blood supply. Aberrant right and left gastric veins, Sappey and Barlow veins, and the parabiliary venous system are considered the most significant examples of third inflow. Clinically, the third inflow is important due to its association with hepatic pseudolesions and its role in increasing surgical complication risks in hepatobiliary and gastric procedures. Our case series includes 8 cases of aberrant left gastric vein (ALGV) and 1 case of a Sappey vein. In addition to presenting examples of ALGV and Suppey vein, we also highlight ischemic complications of the liver observed in a liver transplant donor and a gastric cancer case. Our aim is to emphasize ALGV-related surgical risks and the importance of assessing vascular variations in preoperative imaging.

## Introduction

The term “third inflow” denotes atypical venous drainage to the hepatic parenchyma, occurring outside the conventional dual inflow system of the hepatic artery and portal vein.^1^ Anomalous hemodynamic patterns that deviate from the classic dual supply are associated with liver pseudolesions. Third inflow systems, such as the aberrant left gastric vein (ALGV), epigastric-paraumbilical veins (superior and inferior Sappey veins, Burlow vein), para-biliary venous system, aberrant right gastric vein, and cystic veins, have been described.^2^ Among these variants, ALGV is observed less commonly than the aberrant right gastric vein and the parabiliary venous system.^3^

The ALGV represents an uncommon variation in the stomach’s venous drainage. The incidence of the aberrant LGV was 0.8% in an autopsy study.^4^

**Figure 1.**
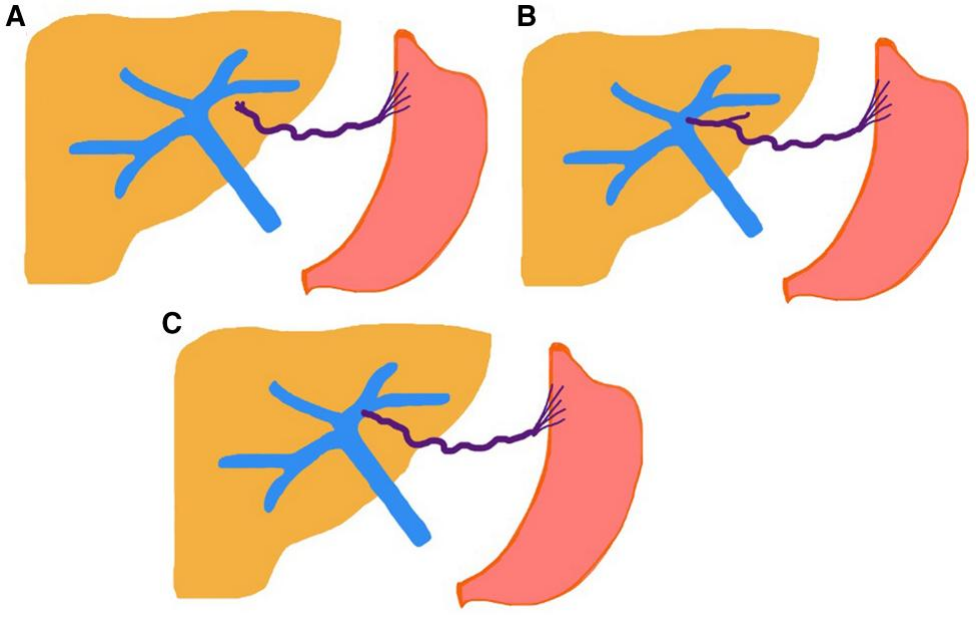
Diagram of aberrant left gastric vein (ALGV) types and their relationship with gastric drainage. (A) Type 1 ALGV drains into the sinusoids in the left hepatic parenchyma. (B) Type 2 ALGV gives off branches that drain into the hepatic parenchyma and ultimately drains into the left portal vein. (C) Type 3 ALGV directly drains into the left portal vein.

In the case of the aberrant left gastric vein variation, the left gastric vein drains into the left portal vein.^3^ Unal (2015) have described 3 different types of ALGV, considering its course and relationship with the left portal vein: Type 1, the left gastric vein drains into the portal sinusoids; Type 2, the left gastric vein drains into both sinusoids and the left portal vein; and Type 3, the left gastric vein directly drains into the left portal vein near the ligamentum venosum (Figure 1).^3^

Hepatic artery variations have been observed in 37% of cases with ALGV.^3^ Therefore, the possibility of coexisting hepatic artery variations should be considered in cases where ALGV is identified.

Preoperative awareness of ALGV is crucial, especially in procedures such as laparoscopic gastrectomy, liver resection, and transplantation, where unrecognized vascular variants may lead to haemorrhage or hepatic ischemia. Frey (2022) provided a surgical perspective on ALGV. In this article, the importance of preoperative recognition of ALGV was discussed.

Hepatic pseudolesions can be defined as areas of parenchyma that show different kinetics in dynamic imaging because they are fed from an additional source other than the normal anatomic supply of the liver. They usually show contrast enhancement in the arterial phase and may be encountered with hypervascular metastases due to this finding. Many hepatic pseudolesions caused by third inflow have been reported in the literature.^2^^,^^3^ Aberrant left gastric vein has been associated with atrophy of segment.^2^^,^^3^ Additionally, its significance has been highlighted in hypertensive gastropathy patients. In a patient with portal hypertension and refractory variceal bleeding despite TIPS placement, an aberrant left gastric vein (ALGV) was identified as the underlying source of recurrent varices. Furthermore, it has been frequently mentioned in the literature as a pathway that could facilitate the early spread of gastric cancer to the liver.

Above, we discussed the potential surgical complications associated with the ALGV, such as pseudolesions and early gastric cancer metastasis in the left liver lobe. However, in certain cases, the presence of the ALGV may provide an advantage to the patient. For instance, in the presence of portal vein thrombosis, the left lobe can continue to receive blood supply through the ALGV. Moreover, in liver transplant candidates with absent splenorenal shunting, the left gastric vein can be considered for portal anastomosis.

Despite the growing body of literature on ALGV and third inflow pseudolesions, their surgical implications remain underrecognized. This study highlights the importance of preoperative identification of the ALGV to prevent complications associated with its unrecognized presence. This study presents 2 postoperative cases with complications attributed to ALGV and several cases in which preoperative identification informed surgical decision-making. One additional case of a liver pseudolesion due to a Sappey vein is also discussed.

### Case 1

**Figure 2.**
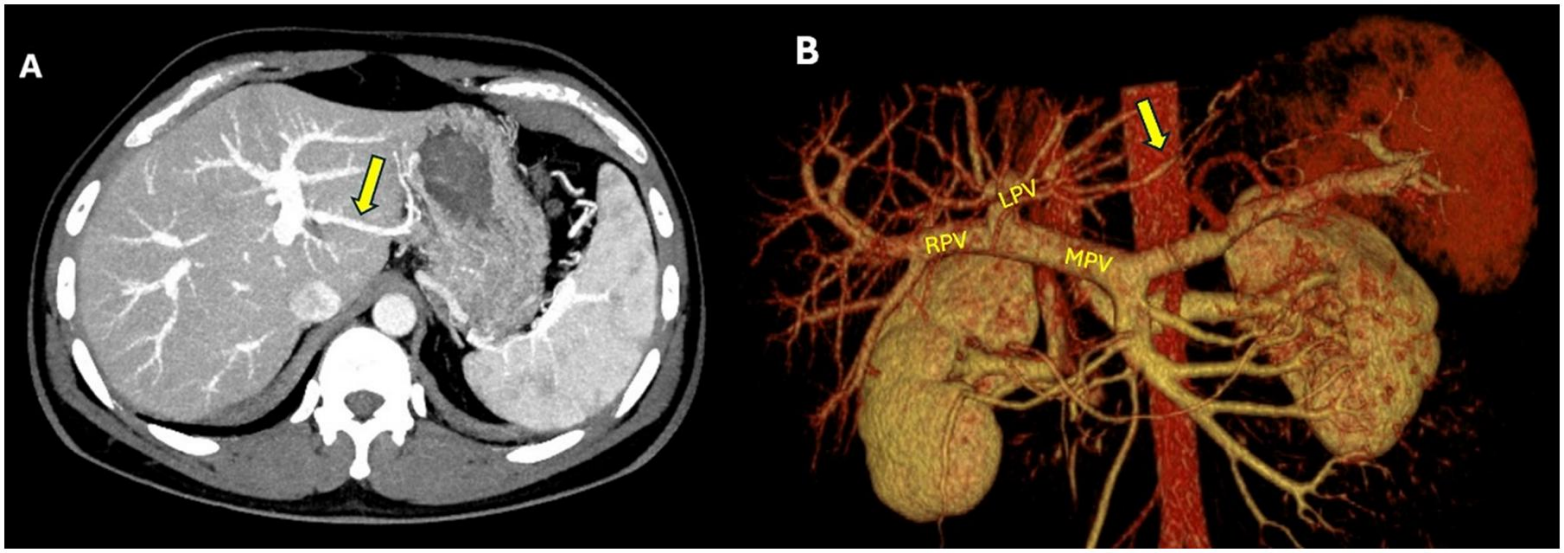
(A) Axial portal phase contrast-enhanced CT maximum intensity projection (MIP) image shows the aberrant left gastric vein draining into the left main portal vein (Type 3) (arrow). (B) 3D reconstruction image of the same patient demonstrates the aberrant left gastric vein draining into the left portal vein (arrow).

A 42-year-old male was evaluated as a potential left lateral segment liver transplant donor at our institution. A routine triphasic abdomen and pelvic CT scan was performed. Preoperative CT scan identified the presence of a Type 3 aberrant left gastric vein (Figure 2). This vein preserved during the harvest procedure. Liver recipient experienced no complications.

### Case 2

**Figure 3.**
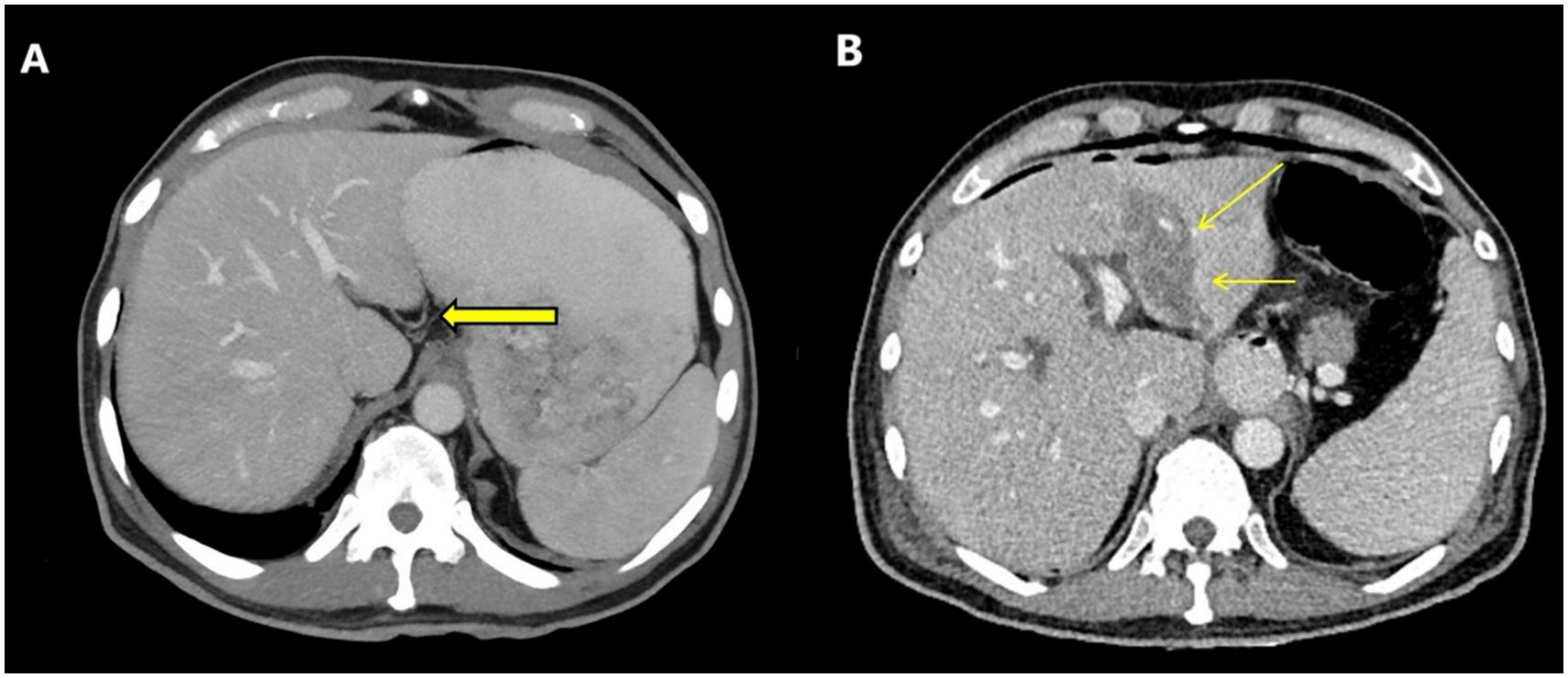
(A) Axial portal phase contrast-enhanced CT MIP image of a preoperative evaluation of a patient with gastric cancer shows the aberrant left gastric vein draining into segment 2 parenchyma (arrow), consistent with Type 1 aberrant left gastric vein. (B) Postoperative abdominal CT of the same patient demonstrates ischemic areas in segments 2, 3, and 4 of the left lobe (arrows).

A 62-year-old male patient presented to our clinic with a preliminary diagnosis of gastric cancer. The preoperative evaluations were performed at an external centre. According to these evaluations, the patient was deemed operable and underwent total gastrectomy and esophagojejunostomy. The patient developed fever and sepsis postoperatively, and an abdominal CT scan revealed triangular ischemic areas in segments 2, 3, and 4A of the liver. Preoperative CT images identified a Type 1 aberrant left gastric vein variation (Figure 3). During surgery, the left gastric vein was ligated as part of the gastrectomy procedure. Alanine transaminase (ALT) and AST levels increased in daily blood tests. The patient was monitored with medical support. Liver function tests began to decline on postoperative day 5, and the patient was discharged. A follow-up examination at 3 months showed regression of the liver findings, although a degree of left lobe atrophy had developed.

### Case 3

**Figure 4.**
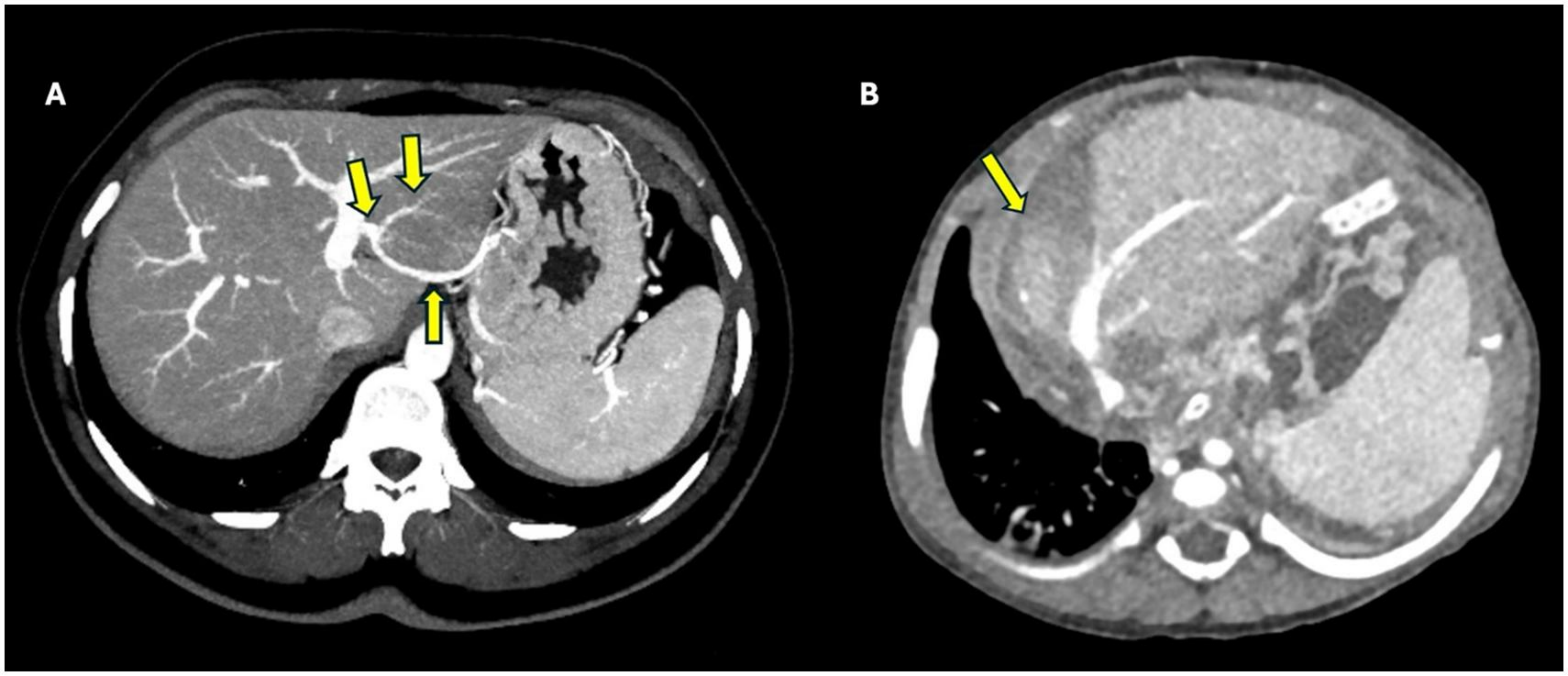
(A) Axial portal phase contrast-enhanced CT MIP image of a preoperative evaluation of a liver transplant donor candidate. The aberrant left gastric vein is seen draining into both the left portal vein and the hepatic parenchyma (arrows). This appearance is consistent with Type 2 ALGV. (B) Axial portal phase contrast-enhanced CT MIP image of a liver transplant recipient (the recipient of the previously mentioned liver transplant donor). In the pediatric recipient, ischemic areas are observed in segment 4.

A 40-year-old female patient presented as a donor candidate for the left lateral segment. Preoperative evaluation revealed a Type 2 aberrant left gastric vein variation (Figure 4). The transplant committee determined that this variation did not pose an obstacle to left lateral segment resection, and the transplant was performed. The left gastric vein was ligated during surgery. No ischaemia-related complications were observed in the postoperative liver function tests. However, the recipient—a 4-month-old infant with biliary atresia and acute cholestatic liver failure who had previously undergone Kasai surgery—developed segment 4 ischemia by postoperative day 1 (Figure 4). Liver enzymes were normalized by day 13. Unfortunately, on postoperative day 18 recipient lost due to multisystemic organ failure.

### Case 4

**Figure 5.**
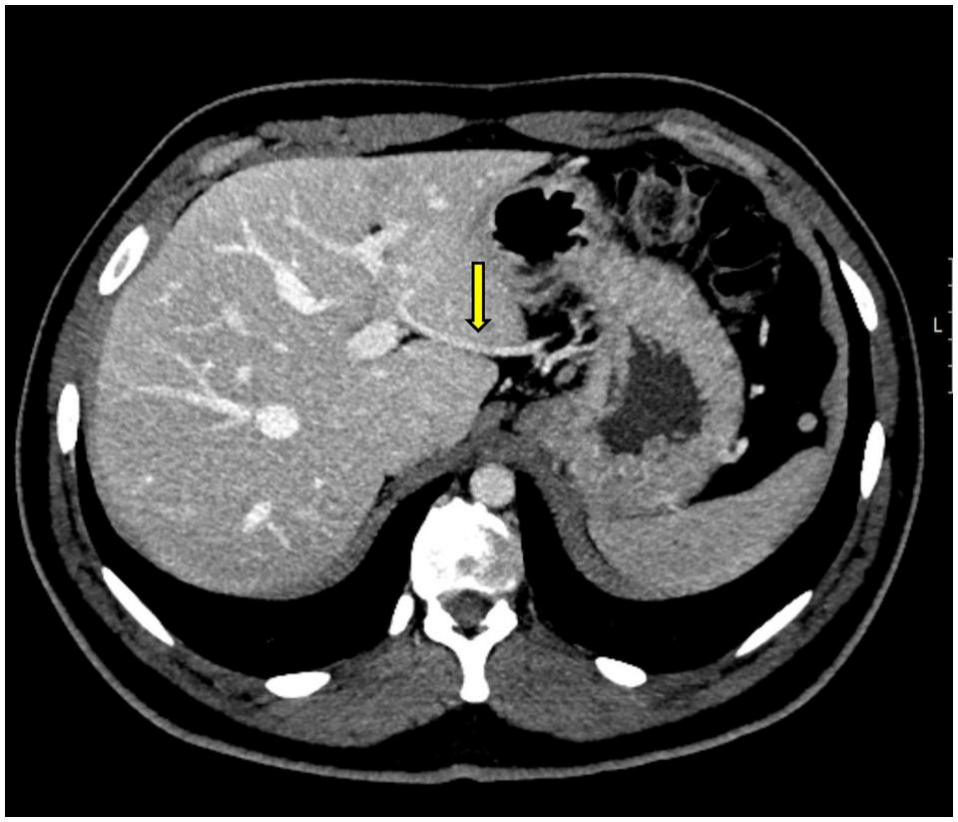
Axial portal phase contrast-enhanced CT MIP image shows the aberrant left gastric vein draining into segment 2 parenchyma (arrow), consistent with Type 1 aberrant left gastric vein.

A 35-year-old male patient, a donor candidate for the right lobe of the liver, was found to have a Type 1 aberrant left gastric vein variation (Figure 5). The surgical team was informed beforehand, and no complications occurred during the surgery.

### Case 5

**Figure 6.**
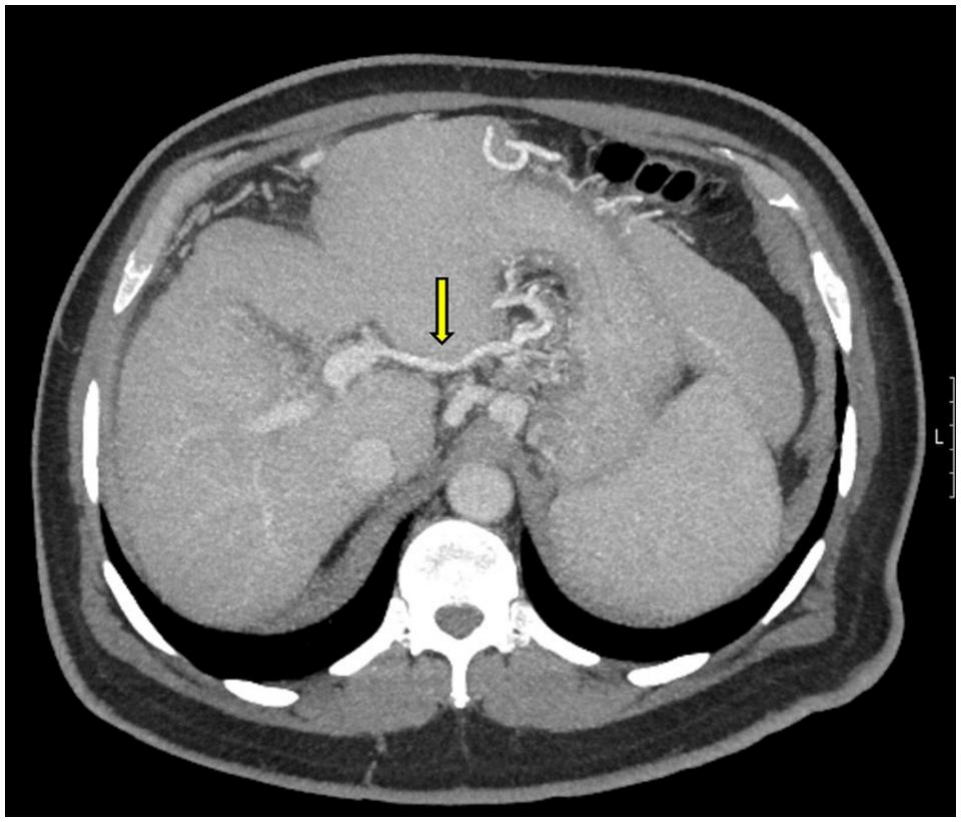
Axial portal phase contrast-enhanced CT MIP image shows the aberrant left gastric vein directly draining into the left portal vein (arrow), consistent with Type 3 aberrant left gastric vein.

A 69-year-old male patient with chronic liver disease (NASH) and alcohol-induced decompensated cirrhosis was evaluated for liver transplantation at our clinic. During the assessment of liver transplant criteria, a triphasic CT scan revealed a Type 3 aberrant left gastric vein variation (Figure 6). The patient had chronic portal vein thrombosis and intra-abdominal collaterals. Due to the patient’s overall status, transplantation was not performed.

### Case 6

**Figure 7.**
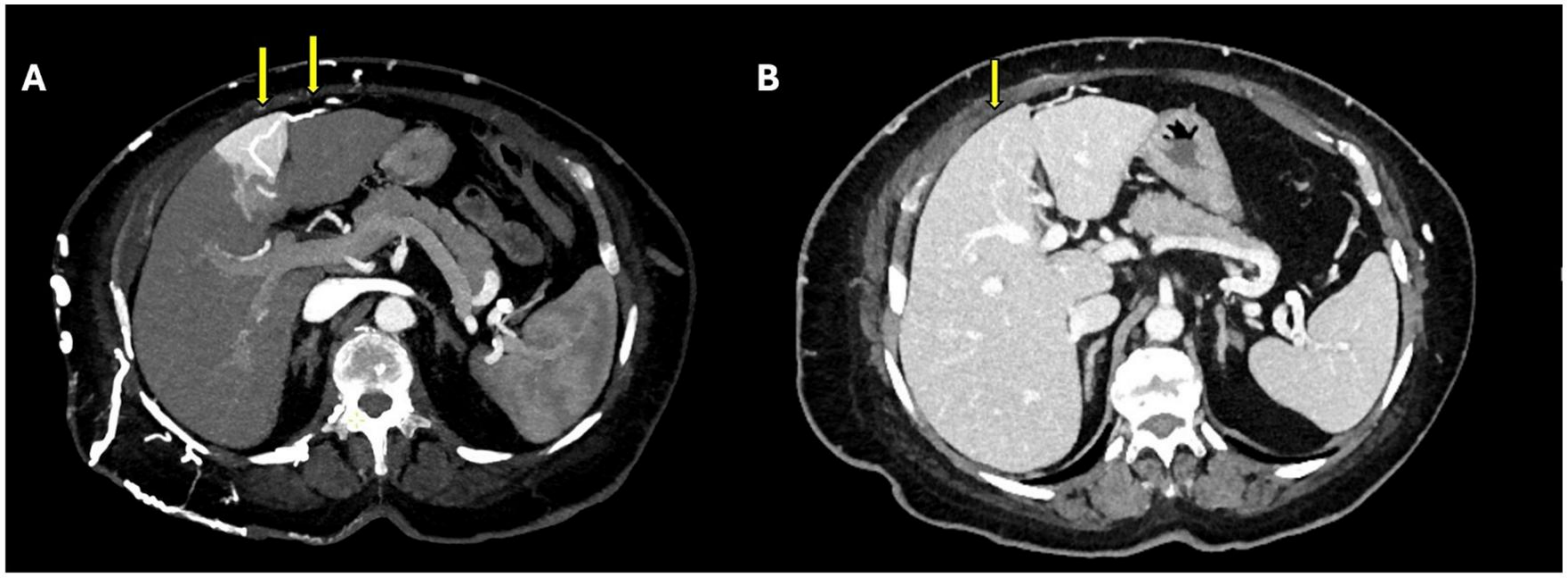
Axial arterial phase (A) and portal phase (B) CT images of a patient under follow-up for renal cell carcinoma. (A) In the arterial phase, the Superior Sappey vein draining into segment 4 parenchyma is observed (arrows). A contrast enhancement area consistent with the “hot spot sign” is visible in segment 4 parenchyma. (B) In the portal phase image, this area appears isodense with the liver parenchyma (arrow). The arterial phase enhancement and isodense appearance in the venous phase are consistent with a pseudolesion.

A 69-year-old female patient with polycystic kidney disease and renal cell carcinoma (RCC) was on hemodialysis following bilateral nephrectomy. A triphasic abdominal CT scan revealed prominent contrast enhancement in segment 4 of the liver during the arterial phase, with vascular structures extending from the abdominal wall into the liver parenchyma. These image findings are consistent with the hot quadrate sign (focal hepatic hot spot sign) (Figure 7). These vascular structures were traced to the internal thoracic vein and were found to be consistent with the superior Sappey vein. It was discovered that the patient had a chronic superior vena cava occlusion. This condition presented as a vascular pseudolesion in segment 4 of the liver. In a patient with a history of RCC, metastasis was considered in the differential diagnosis of a lesion with prominent arterial contrast enhancement. Differentiating between a pseudolesion and RCC metastasis was essential for the management of the patient.

### Case 7

**Figure 8.**
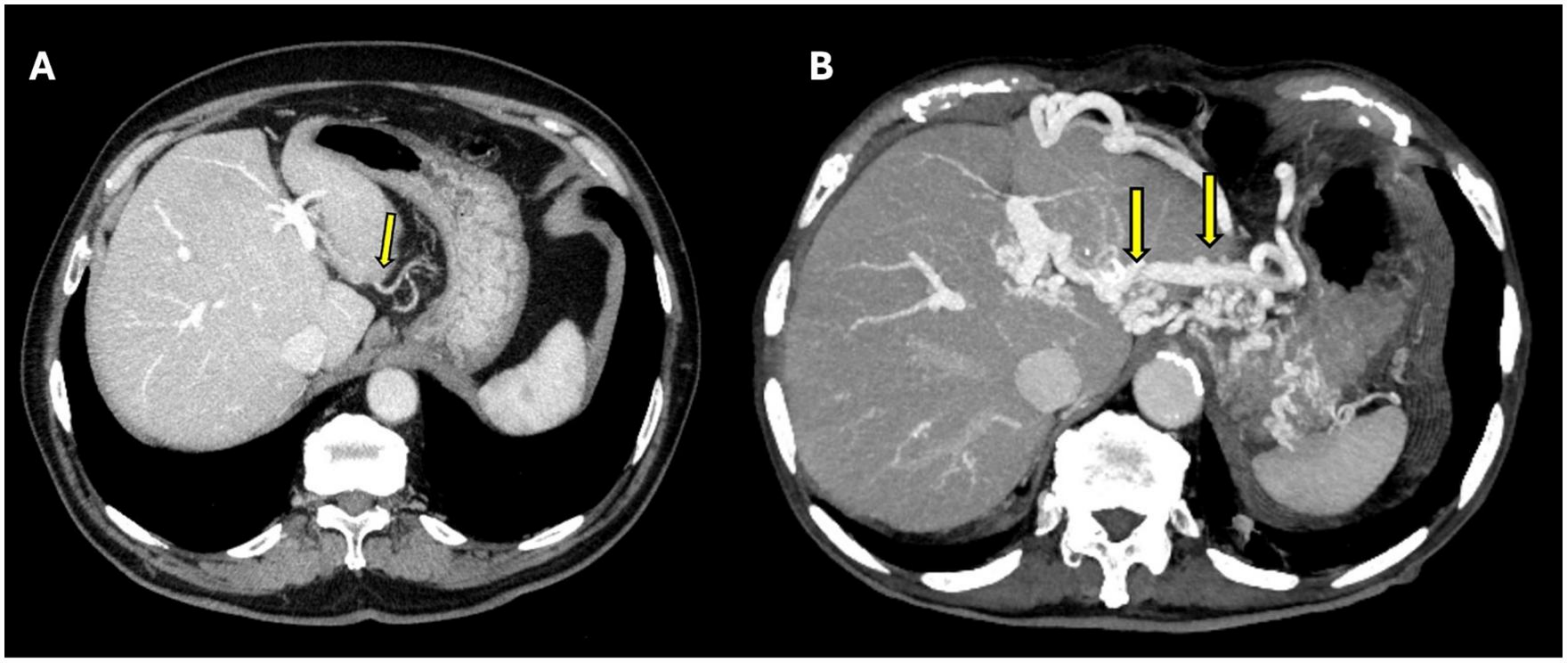
(A) Axial portal phase contrast-enhanced CT MIP image of a patient evaluated for pancreatic cancer demonstrates the aberrant left gastric vein draining into both segment 2 parenchyma and the left portal vein (arrow), consistent with Type 2 ALGV. (B) In the follow-up CT MIP image 1 year later, a collateral vessel secondary to pancreatic tumour invasion into the portal vein is observed. The development of collateral vessels makes Type 2 ALGV more apparent (arrows).

An 82-year-old female patient was referred to our clinic with a preliminary diagnosis of pancreatic neoplasia. Triphasic CT revealed a hyperenhancing mass invading the portosplenic confluence. While evaluating venous invasion, a Type 2 aberrant left gastric vein variation was identified. Collaterals had developed secondary to portal vein invasion in this case (Figure 8). Varices around the stomach, a common finding in portal hypertension, were delayed in this case due to the left gastric vein draining into the left portal vein.

### Case 8

**Figure 9.**
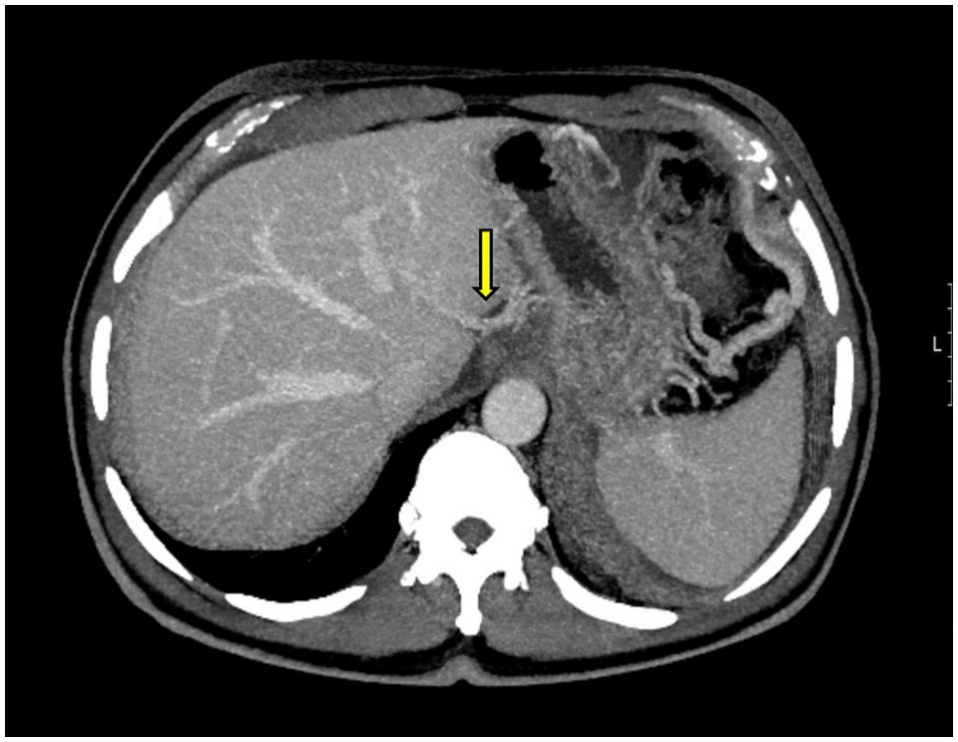
Axial portal phase contrast-enhanced CT MIP image shows the aberrant left gastric vein draining into both segment 2 parenchyma and the left portal vein (arrow), consistent with Type 2 ALGV.

A 61-year-old male patient with a diagnosis of follicular-type Non-Hodgkin lymphoma was under follow-up. Abdominal imaging revealed chronic pancreatitis and secondary SMV thrombosis. The patient was found to have a Type 2 aberrant left gastric vein variation (Figure 9). This anatomical variant may have offered protective venous drainage, minimizing complications from pancreatic enzyme leakage typically directed toward the portal vein confluence.

### Case 9

**Figure 10.**
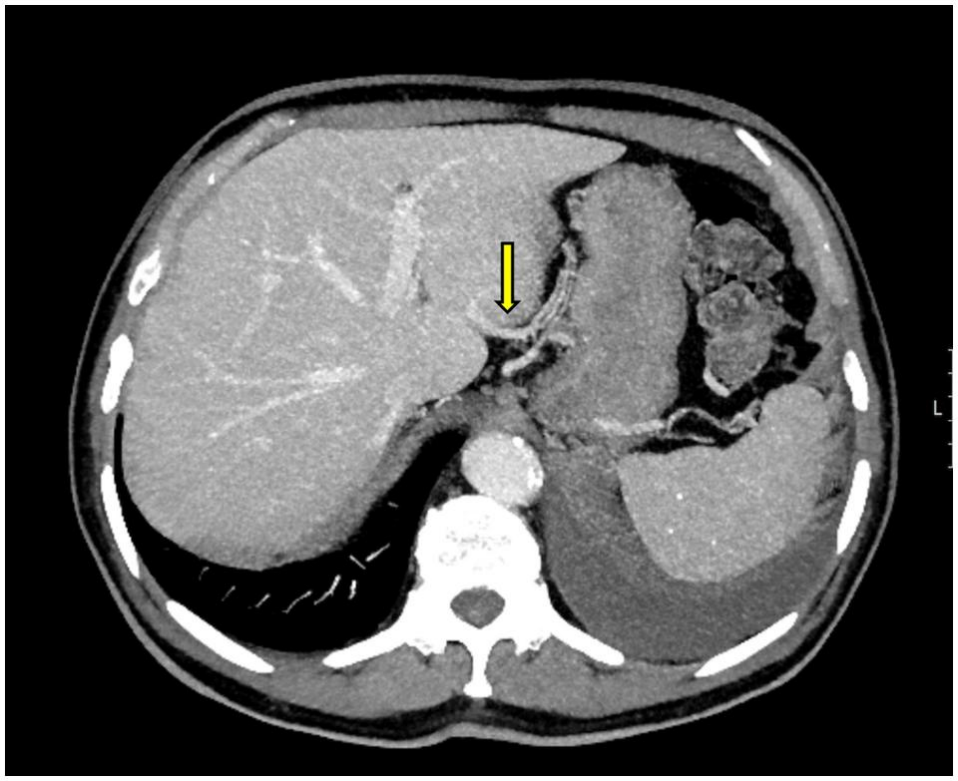
Axial portal phase contrast-enhanced CT MIP image shows the aberrant left gastric vein draining into segment 2 parenchyma (arrow), consistent with Type 1 ALGV.

A 74-year-old male patient with a history of leukaemia is under follow-up for lung cancer. Abdominal imaging revealed widespread intra-abdominal metastases, and a Type 1 ALGV variation was also identified (Figure 10).

## Discussion

This case series includes right liver lobe and left lateral segment liver donor candidates, a liver transplant recipient candidate, a patient with gastric cancer, and other patients under oncological follow-up. These patients underwent left lateral segmentectomy, right hepatectomy, and total gastrectomy surgeries. In the patient who underwent total gastrectomy, ischaemia developed in the left lobe of the liver in the postoperative period secondary to the ligation of the ALGV. In contrast, in transplant recipients and donors, preoperative identification and surgical awareness of ALGV helped prevent complications.

Additionally, 1 patient had a pseudolesion appearance in the liver due to the development of a Sappey vein. Similar cases have been reported in the literature.^5^^,^^6^

In our case series, Type 1 and Type 2 were observed with equal frequency, each accounting for 37.5%; Type 3 was the least common type, observed in 25% of cases.

In the study by Unal (2019), which identified 32 cases of ALGV, type 1 ALGV was reported as the most common type.^7^ Choi (2020), in a study identifying 21 ALGV cases, utilized a different classification system.^8^ According to Unal’s classification, Choi’s study reported 28.6% of cases as types 1 and 2 and 71% as type 3.^8^ In our case series, the results may have differed due to the limited number of patients. Additionally, this article presents case examples independent of a retrospective screening methodology.

Recognition of vascular variants is essential during preoperative imaging to mitigate surgical risk. Variations in the portal venous and hepatic arterial systems are common and must be evaluated, particularly in oncologic patients, where both tumour invasion and baseline anatomy can affect surgical planning. Evaluating venous variants is especially challenging in the hepatic hilum, where the differentiation of arteries and veins can be difficult on venous-phase imaging alone. Although complex venous drainage variations may not seem critical in routine evaluations, as seen in our cases, they are of vital importance in preoperative planning for organ resections. Awareness of these variations and incorporation of this knowledge into radiology reporting may prevent serious postoperative complications. Our experiences, along with the cases reported in the literature, underscore the importance of radiologists being more familiar with the aberrant left gastric vein variation.

## Conclusion

Radiologists should be aware that the left gastric (coronary) vein, a component of the liver’s third inflow, may demonstrate variable drainage patterns and should be systematically evaluated in preoperative imaging.

## Learning points

- The third inflow is a relatively unknown phenomenon that may result in hepatic pseudolesions. Familiarity with third inflow pathways is essential to prevent diagnostic errors and unnecessary interventions.
- Aberrant left gastric vein is an example of a third inflow. It can be a cause of complications in hepatic and gastric surgeries. Therefore, radiologists should pay attention to this anatomical variation during preoperative planning.
